# Proposed changes to the ACR phantom filling procedure for more accurate and consistent activity concentrations

**DOI:** 10.1002/acm2.12531

**Published:** 2019-01-16

**Authors:** Bradley J. Beattie

**Affiliations:** ^1^ Memorial Sloan Kettering Cancer Center New York NY USA

**Keywords:** ACR phantom, PET image harmonization, quality assurance

## Abstract

**Objectives:**

The phantom filling procedures currently specified by the American College of Radiology (ACR) for its PET accreditation program unnecessarily limit how tight the tolerances can be made on the accuracy requirements for the concentrations measured in the resultant images.

**Methods:**

New procedures are proposed to improve the accuracy and consistency of the concentrations within the phantom at the time of imaging. These improvements are gained by exchanging the difficult process of accurately measuring a dose with the more easily achieved accurate measurements of time and liquid volume to control final radioactivity concentrations. A comparison of the results when following the two filling procedures is made.

**Results:**

The variability in metrics specified by the ACR was approximately halved by following the new procedures.

**Conclusion:**

These improvements allow tighter thresholds to be applied when evaluating image quality and quantitative accuracy of the PET images. These changes also render this phantom data more suitable for inter‐PET‐scanner harmonization and improve its utility for comparing image reconstruction methods.

## 1 INTRODUCTION

The American College of Radiology (ACR) has been accrediting medical imaging programs since 1987. Such programs perform an important service providing peer review of a facility's staff qualifications, imaging equipment, quality control procedures and ultimate image quality. As part of their nuclear medicine accreditation program, the ACR has published detailed instructions describing the acquisition and analysis of PET phantom data to be submitted for review by the ACR during the accreditation process. These instructions specify the phantom to be used (an Esser PET Phantom with a Flangeless Deluxe Jaszczak Lid) along with specific target fluorine‐18 radioactivity concentrations based on the injected dosage typically used by the site. The sites are also instructed to use their standard protocols for image acquisition and reconstruction. The overall goal of the phantom filling instructions is to achieve specific levels of radioactivity both within the main chamber and within the cylindrical subregions of the phantom at the time of imaging. However, because the instructions specify a fixed dilution volume and assume that the two main dose measurements are made simultaneously, the accuracy of the radioactivity concentrations achieved is unnecessarily limited.

The intent in providing this level of detail is to achieve radioactivity concentrations that are consistent with [^18^F]FDG concentrations seen in patients at the time of imaging. The absolute level of radioactivity is important because the signal‐to‐noise ratio in PET data is not only a function of the counting noise associated with the true coincidences but also on the noise introduced by the subtraction of the scatter and random coincidences. In particular, the noise stemming from the random coincidences are heavily dependent on the absolute amount of radioactivity present, this is because the number of random coincidences increases quadratically with radioactivity whereas true coincidences vary linearly with radioactivity (specifically the number of positron emissions). Thus, in establishing a reference for the image noise level encountered within a given PET imaging system, it is imperative that the radioactivity at the time of imaging be considered and carefully controlled.

The ACR accreditation instructions set target amounts of radioactivity that are proportional to the patient doses used by the candidate institution for a clinical whole‐body FDG PET scan. For example, if a given institution would typically give a 10 mCi dose to a patient, then the ACR instructions are to fill the main “background” chamber of the phantom with 830 uCi of F‐18 and to fill the “hot” cylinders with radioactivity having a concentration of 350 nCi/ml (350 uCi diluted in 1000 ml). The instructions also stipulate that the phantom should be imaged precisely 60 min after the background dose is measured, much like the recommended delay following the patient's ^18^F‐FDG injection. Moreover, it is specified that the acquisition protocol and reconstruction parameters match that which is typically used at the institution for a standard whole‐body study.

If consistent radioactivity levels can be achieved, the reconstructed images of this phantom can serve as a reference via which interscanner and interinstitutional comparisons of image quality can be made. The quality of (and noise within) the images does not directly describe the quality and noise of a patient image, but one can roughly predict that if an institution following a given set of clinical procedures produces higher quality phantom images than that of another institution following different procedures or with a different PET camera system, then their patient images will likely be better as well (assuming neither is beyond their camera's peak noise equivalent count rate during their patient imaging). Similarly, these images can serve as references (although imperfect ones) when attempting to harmonize image quality between PET camera systems through adjustments in dose, acquisition time, and reconstruction parameters.

In addition to facilitating these intercamera comparisons, the ACR phantom serves as a routine check of the quantitative accuracy of the camera system. By following the recommended procedures (which includes specifying a 70 kg patient injected with the standard dose used at the facility), the expected background radioactivity should have an SUV of unity. The ACR‐specified tolerance on this background level (tellingly) is a generous ±15%. The anticipated variability implicit in this specification is significantly greater than one would normally expect for a repeated measurement of a large region of background radioactivity and it is the thesis of this manuscript that this and other tolerances could be made much tighter if certain procedural deficiencies were to be addressed.

The difficulty stems from the need, when following the ACR procedures, to precisely draw up the specified ^18^F doses, especially given the delay between the two measurements. Measuring a dose drawn into a syringe generally involves a series of radioactivity measurements with small adjustments in the amount of radioactivity between each. If it is too low, more is added. If it is too high, radioactivity is discarded. Any error in the measurement of the first dose (“dose A” in ACR's parlance) will directly result in an error in the magnitude of the measurements taken from the hot cylinders. Any error in either the timing or the magnitude of the second dose (“dose B”) will directly be reflected as an error in the SUV of background activity at the time of imaging. No adjustment is made within the ACR instructions to account for the time that it takes to get the second dose correct. If this measurement is for some reason delayed or takes an extended time to get right, then this will result in errors in both the absolute activity in the cylinders and in contrast relative to the background. This uncertainty in the true contrast is particularly problematic when seeking to assess image quality using contrast‐to‐noise and related metrics.

## 2 METHODS

Two simple modifications of the ACR phantom filling procedure can improve the accuracy of the radioactivity levels achieved within the phantom. Specifically, I propose that dose B should be measured first and need only be approximate. The dose should still be measured precisely, along with a precise recording of the time of measurement, but any radioactivity near the target level is acceptable. Instead, the time delay, *t* (in minutes), before imaging is adjusted to achieve the desired radioactivity concentration in the background at the time of imaging, using the formula shown in eq. (1).

(1) $$t=-T_{1/2}log_{2}\left(\frac{d_{t}\cdot 2^{\left(-60/T_{1/2}\right)}}{d_{m}}\right),$$

where *d*_*t*_ is the target dose (e.g., 350 uCi), *d*_*m*_ is the (approximate) dose that was actually measured and *T*_*1/2*_ is the half‐life of ^18^F in minutes (i.e., 109.771 min). Note that the constant, 60, in the formula refers to the original specified delay time in minutes.

It is also worth noting that by purposefully increasing or decreasing *d*_*m*_, it is possible to reduce or increase the wait time for your convenience. For simplicity and clarity, it is assumed here that the entire drawn dose can be transferred into the background of the phantom with negligible residual (something generally easily achievable with a few flushes of the syringe). If this is not the case, adjustments to the formula can be made to compensate for the residual radioactivity.

Following the measurement and transfer of dose B into the phantom, dose A can once again be drawn up approximately and without significant time pressure. In this instance, the desired concentration (at the newly designated time of imaging) will be achieved by controlling the dilution volume, *v*, according to the formula shown in eq. 2.

(2) $$v=1000\cdot \frac{d_{m}}{d_{t}}\cdot 2^{\left(\frac{60-\left(t_{i}-t_{m}\right)}{T_{1/2}}\right)},$$

where *t*_*i*_ *‐t*_*m*_ is the difference in minutes between the target imaging time and the time at which dose A was measured. And where *d*_*m*_ is again the approximately measured dose (this time of dose A) and *d*_*t*_ is the target dose A specified by the ACR. Note that here the constant, 1000, refers to the original dilution volume specified by the ACR in milliliters.

It is also again worth noting that by purposely reducing the measured dose *d*_*m*_, it is possible to reduce the volume of radioactivity generated (we typically try for about 250 ml) to avoid having to store or dispose of a large volume of radioactive liquid. The reductions in volume, however, should not be pushed too far in order that sufficient volume is available to fill all the cylinders and also to maintain the high percent accuracies associated with measuring large volumes with (for example) a graduated cylinder. In addition to a graduated cylinder, a container to mix the dose and a spreadsheet to perform the calculations are helpful when performing these procedures.

By following this procedure, it is expected that the ACR specified radioactivity concentrations within background and hot cylinders of the Esser phantom can be achieved both more accurately and with greater consistently than is possible when following the ACR specified procedure. To test this hypothesis, both phantom filling procedures (the proposed and the original) were tried during the quarterly QA at my institution, on the same PET camera (a GE D690) but at different times. The quantitative accuracy and consistency of the resultant images were then compared.

## 3 RESULTS

The ACR target SUV levels in the background and hot cylinder regions of the phantom are 1.0 and 2.45, respectively. At my institution we routinely use a 12 mCi patient injection. This corresponds to activity concentrations at the time of imaging for the phantom of 117.4 nCi/ml and 287.5 nCi/ml, respectively. The ACR analysis procedures stipulate that two metrics, the background mean SUV and the SUVmax of the 25 mm diameter hot cylinder, be reported. The tolerances on these metrics prescribed by the ACR are 0.85–1.15 and 1.8–2.8, respectively. While following the ACR prescribed filling method during the quarterly QA over a period of 2 yr (i.e., eight times total) the coefficient of variance about the expected value was 5.1% for the ACR specified mean of the background region and 7.2% for the max value within the hot cylinder. The ACR specified tolerance for the mean SUV is therefore consistent (approximately) with a three‐sigma variation about the target value. The ACR specified tolerance on the SUVmax of the hot cylinder, however, is quite asymmetric ranging from 26.5% below to 14.3% above the target value. This presumably is to allow for reductions in the max value owing to high partial voluming on (i.e., low resolution of) some scanners. Based on the variability seen in this set of phantoms, the tolerance corresponds to a −3.7 to +2.0 sigma acceptable range.

When following the newly proposed phantom filling procedure (again eight trials over a 2‐yr period) the variances measured went down to 2.5% for the background mean and to 4.1% for the cylinder maximum, approximately half the variability seen when following the ACR instructions. These improvements allow tighter thresholds to be applied when evaluating both image quality and quantitative accuracy of the PET images.

## 4 CONCLUSION

The phantom specified for ACR accreditation has several potential uses beyond accreditation including the harmonization of image quality across PET camera systems^1^ and for optimization of reconstruction algorithms and parameters.^2^ However, to best serve these purposes it is important that the activity concentrations within the various sub‐regions of the phantom are both accurate and precise. Using the phantom filling procedures recommended by the ACR it is difficult to achieve the requisite level of accuracy and consistency. In the filling procedures proposed here, the need to draw into a syringe a precise amount of radioactivity is removed and instead time and dilution (both of which can be controlled very precisely) is used to determine the final activity concentrations. Although the degree of improvement to be gained following these new procedures is in large part dependent on the care taken when following the ACR recommend procedure, the new procedure is faster, easier and results in accuracies near the limit of the combined variability in the PET and dose calibrator hardware.

## CONFLICT OF INTEREST

I have no conflicts of interest related to this work.
